# Application of transfer learning to predict drug-induced human in vivo gene expression changes using rat in vitro and in vivo data

**DOI:** 10.1371/journal.pone.0292030

**Published:** 2023-11-30

**Authors:** Shauna D. O’Donovan, Rachel Cavill, Florian Wimmenauer, Alexander Lukas, Tobias Stumm, Evgueni Smirnov, Michael Lenz, Gokhan Ertaylan, Danyel G. J. Jennen, Natal A. W. van Riel, Kurt Driessens, Ralf L. M. Peeters, Theo M. C. M. de Kok

**Affiliations:** 1 Maastricht Centre for Systems Biology (MaCSBio), Maastricht University, Maastricht, The Netherlands; 2 Dept. of Biomedical Engineering, Eindhoven University of Technology, Eindhoven, The Netherlands; 3 Eindhoven Artificial Intelligence Systems Institute (EAISI), Eindhoven University of Technology, Eindhoven, The Netherlands; 4 Dept. of Advanced Computing Sciences, Maastricht University, Maastricht, The Netherlands; 5 Institute of Organismic and Molecular Evolution, Johannes Gutenberg University Mainz, Mainz, Germany; 6 Preventive Cardiology and Preventative Medicine – Center for Cardiology, University Medical Center of the Johannes Gutenberg University Mainz, Mainz, Germany; 7 Sustainable Health, Flemish Institute for Technological Research (VITO), Mol, Belgium; 8 Dept. of Toxicogenomics, GROW School for Oncology and Reproduction, Maastricht University, Maastricht, The Netherlands; IBU: International Burch University, BOSNIA AND HERZEGOVINA

## Abstract

The liver is the primary site for the metabolism and detoxification of many compounds, including pharmaceuticals. Consequently, it is also the primary location for many adverse reactions. As the liver is not readily accessible for sampling in humans; rodent or cell line models are often used to evaluate potential toxic effects of a novel compound or candidate drug. However, relating the results of animal and *in vitro* studies to relevant clinical outcomes for the human *in vivo* situation still proves challenging. In this study, we incorporate principles of transfer learning within a deep artificial neural network allowing us to leverage the relative abundance of rat *in vitro* and *in vivo* exposure data from the Open TG-GATEs data set to train a model to predict the expected pattern of human *in vivo* gene expression following an exposure given measured human *in vitro* gene expression. We show that domain adaptation has been successfully achieved, with the rat and human *in vitro* data no longer being separable in the common latent space generated by the network. The network produces physiologically plausible predictions of human *in vivo* gene expression pattern following an exposure to a previously unseen compound. Moreover, we show the integration of the human *in vitro* data in the training of the domain adaptation network significantly improves the temporal accuracy of the predicted rat *in vivo* gene expression pattern following an exposure to a previously unseen compound. In this way, we demonstrate the improvements in prediction accuracy that can be achieved by combining data from distinct domains.

## Introduction

Drug-induced liver injury is a leading cause of the failure of novel candidate drugs during end-stage clinical trials [1]. This drug-induced liver injury occurs despite the compound having already successfully undergone a battery of costly and time-consuming tests prior to human testing. The current gold standard for evaluating a compound for potential adverse outcomes is the two-year rodent *in vivo* bioassay. However, a survey published in 2000 reported that just 43% of the toxic effects of pharmaceutical compounds in humans were correctly predicted by tests in rodents [2]. Moreover, the two-year rodent *in vivo* bioassay is expensive, time-consuming, and necessitates the sacrifice of large numbers of animals to screen a single compound. The apparent lack of sensitivity and specificity in predicting potential adverse outcomes in humans, coupled with growing ethical concerns surrounding animal testing, has motivated the development of alternatives to the traditional rodent *in vivo* bioassays, most notably *in vitro* cellular models [3]. These *in vitro* toxicogenomics approaches often use human cell lines, eliminating the need for animal testing while aiming to provide a more relevant prediction of adverse effects for the human system.

Multiple studies have reported promising results in differentiating between subclasses of carcinogenicity [4, 5] and predicting hepatotoxicity of a novel compound using genomic signatures of human *in vivo* disease states [6–13]. Nevertheless, *in vitro* assays are not without their limitations. These cell-line models lack the systemic interplay with other tissues that exist *in vivo*. Consequently, the *in vitro* models may differ in functionality and metabolism from the tissues they represent. Moreover, immortalised human cell lines, such as hepatic HepaRG and HepG2 cells, are frequently used in toxicity testing as they continue to grow and divide indefinitely *in vitro* [14]. However, these cell lines are often tumour-derived, and liver-specific metabolic functions tend to vanish as culture time increases [15, 16]. Therefore, genomic signatures obtained from these cell lines following exposure to a compound may not necessarily reflect human *in vivo* disease states, particularly if we are trying to go beyond the simple classification of potential toxicants and derive mechanistic insight into modes of action of toxicity. Consequently, there is a need for new methods that can better relate the output from these *in vitro* exposure assays to potentially relevant human *in vivo* disease states.

A recent study has reported notable success in applying deep learning architectures to translate time series of hepatic gene expression following an exposure from one domain to another, predicting both human *in vitro* and rat *in vivo* gene expression patterns in response to a previously unseen compound given a measured time series of rat *in vitro* gene expression [17]. However, training deep learning models require large volumes of data. While large databases containing both rat *in vitro* and rat *in vivo* hepatic gene expression following exposures to a vast array of compounds are available [18, 19], human *in vivo* data is comparatively sparse as the liver is not readily accessible for sampling. Consequentially, insufficient human *in vivo* data is available to effectively train a deep learning model such as those used by O’Donovan et al. [17] for human *in vivo* gene expression predictions.

In many situations, it may be too laborious, costly, or even infeasible to obtain sufficient data to train a reliable predictor. Transfer learning is a branch of machine learning in which knowledge gained from solving one problem is re-used while solving another similar problem [20]. Multiple studies have applied a range of transfer learning or domain adaptation algorithms to the cross-species prediction task [21–24] with mixed success. Transfer learning approaches have been applied to translate gene expression measured at a single time point from clinical *in vitro* models to primary human tumour profiles to better predict the mutation status of the tumour [25] or predict a patient’s response to a particular treatment [26]. Ganin et al. proposed a method to integrate domain adaptation and deep feature prediction in the context of a generalisable neural network architecture [27]. This method allows a predictor model to be trained for an unlabelled target data set using a large, similarly distributed, labelled data set. Ganin et al. showed their unsupervised domain adaptation method outperformed the state-of-the-art in image processing and sentiment analysis of natural language [27].

In this study, we integrate domain adaptation within previously validated deep learning architectures [17] using the method proposed by Ganin et al. We then apply the resulting domain adaptation network to leverage a large publicly available data set of measured rat *in vitro* and *in vivo* gene expression following an exposure to a range of compounds from Open TG-GATEs to facilitate the training of a predictor model of human *in vivo* gene expression. We also evaluate the impact of incorporating the human *in vitro* data in the rat *in vitro* to rat *in vivo* prediction. Finally, we explore the potential of the reduced dimensional representation of the data generated by the bottleneck architecture of our deep neural network to classify compounds based on toxicity.

## Materials and methods

### Open TG-GATEs

**Fig 1:**
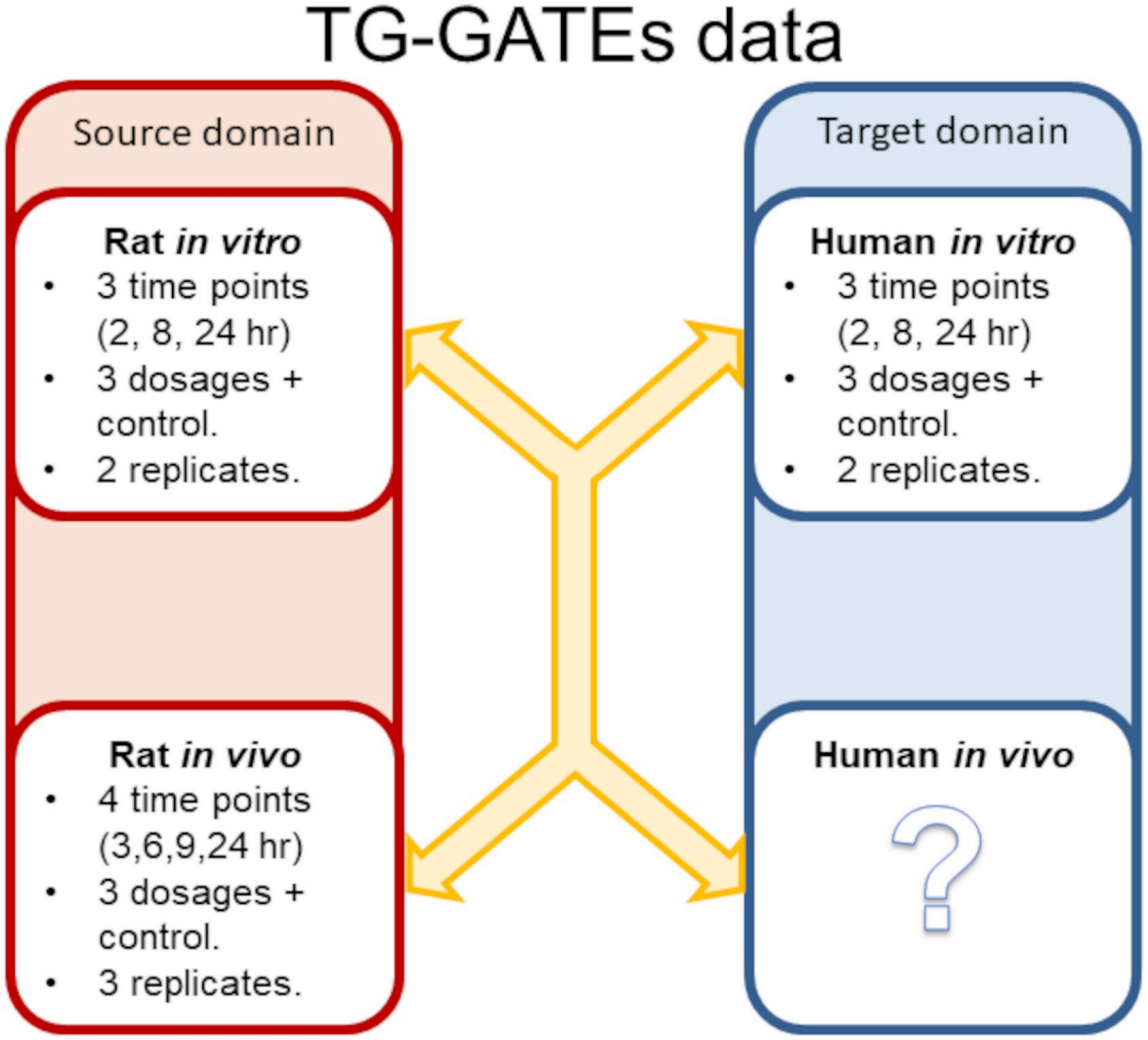
Overview of toxicogenomics data from TG-GATEs included in this study. Overview of toxicogenomics measurements available from the TG-GATEs database used in this study. Gene expression profiles were measured for primary rat and primary human hepatocytes exposed *in vitro* at three time points (2, 8, and 24 hours) following exposure to 45 compounds at three dosages (low, medium, and high) plus a control. Two biological replicates were performed for each compound-dosage combination. Gene expression profiles were measured at four time points (3, 6, 9, and 24 hours) for rat hepatic tissue exposed *in vivo* to 45 compounds at three dosages (low, medium, and high) plus a control. Three biological replicates are conducted for each rat *in vivo* exposure. The model is trained to predict *in vivo* gene expression profile following exposure to a compound given a time series of *in vitro* data using the rat data (labelled source domain) and through the use of domain adaptation the model can also be used to predict human *in vivo* gene expression given a measured time series of human *in vitro* gene expression (unlabelled target domain).

Open TG-GATEs is a large publicly available toxicogenomics database containing gene expression profiles from *in vitro* assays in both primary rat and primary human hepatocytes and *in vivo* rats following exposure to 170 compounds [18]. For the *in vitro* exposures, gene expression profiles were measured at three time points (2, 8, and 24 hours) following a single exposure to a given compound at three dosages (low, medium, and high) plus control, with two biological replicates for each compound-dose combination. For the rat *in vivo* experiments, gene expression profiles were measured at four time points (3, 6, 9, and 24 hours) following a single exposure to a compound at a low, medium, and high dosage plus a control (Fig 1). Gene expression profiles for the rat *in vitro* and *in vivo* samples were generated using the Affymetrix Rat Genome 230 2.0 Array, and human *in vitro* gene expression profiles were measured using the Affymetrix Genome U133 Plus 2.0. Array data for rat and human *in vitro* and rat *in vivo* exposures for all compounds were downloaded in the form of CEL files from the Open TG-GATEs database (https://toxico.nibiohn.go.jp) and pre-processed using Affymetrix Power Tools using the robust multi-array average normalisation method. Following normalisation, compounds missing either time points or dosages were removed, leaving a data set of 45 compounds with a complete set of measurements for use in this study (S1 Table in S2 File).

### Learning examples

The rat *in vitro* and *in vivo* data form the source domain and will be used to train a predictor model for the unlabelled human *in vitro* target domain (Fig 1). Learning examples for the source domain are generated by pairing the time series of gene expression values for a specific compound-dose combination for the rat *in vitro* data with a time series of gene expression values for the same compound-dose combination in the rat *in vivo* data, as described previously [17]. To maintain the structure of the data in each domain (human and rat *in vitro* and rat *in vivo*) one of the three rat *in vivo* replicates is discarded as described in [17]. As each rat *in vitro* biological replicate for a given compound-dose combination is a valid match for both rat *in vivo* replicates four learning examples can be generated for each compound-dose combination. With 45 compounds, three dosages plus the control and the pairwise matching of replicates 720 labelled rat *in vitro* to *in vivo* learning examples are generated for the source domain. The human *in vitro* data (the target domain) is processed in the same manner as the rat *in vitro* data producing 720 unlabelled human *in vitro* learning examples.

#### Gene sets

Microarray data measures the expression of more than 20,000 gene transcripts, resulting in a high dimensional feature space. While Open TG-GATEs is a comparatively large database of toxicogenomics data, the 720 labelled learning examples that can be generated from the data are insufficient to effectively train a model that could predict genome-wide gene expression. Consequently, it was decided to restrict our analyses to four subsets of genes reported in literature as being associated with relevant toxicological outcomes [17] namely gene sets linked to steatosis (developed in-house from the KEGG pathway hsa04932, S3 Table in S2 File), cholestasis [28–30], genotoxicity and carcinogenicity (GTX+C) [6, 7, 31], and non-alcoholic fatty liver disease (NAFLD) [32]. All gene lists are filtered to contain only known rat-human orthologs. Complete gene sets are listed in S2-S5 Tables in S2 File.

#### Model

**Fig 2:**
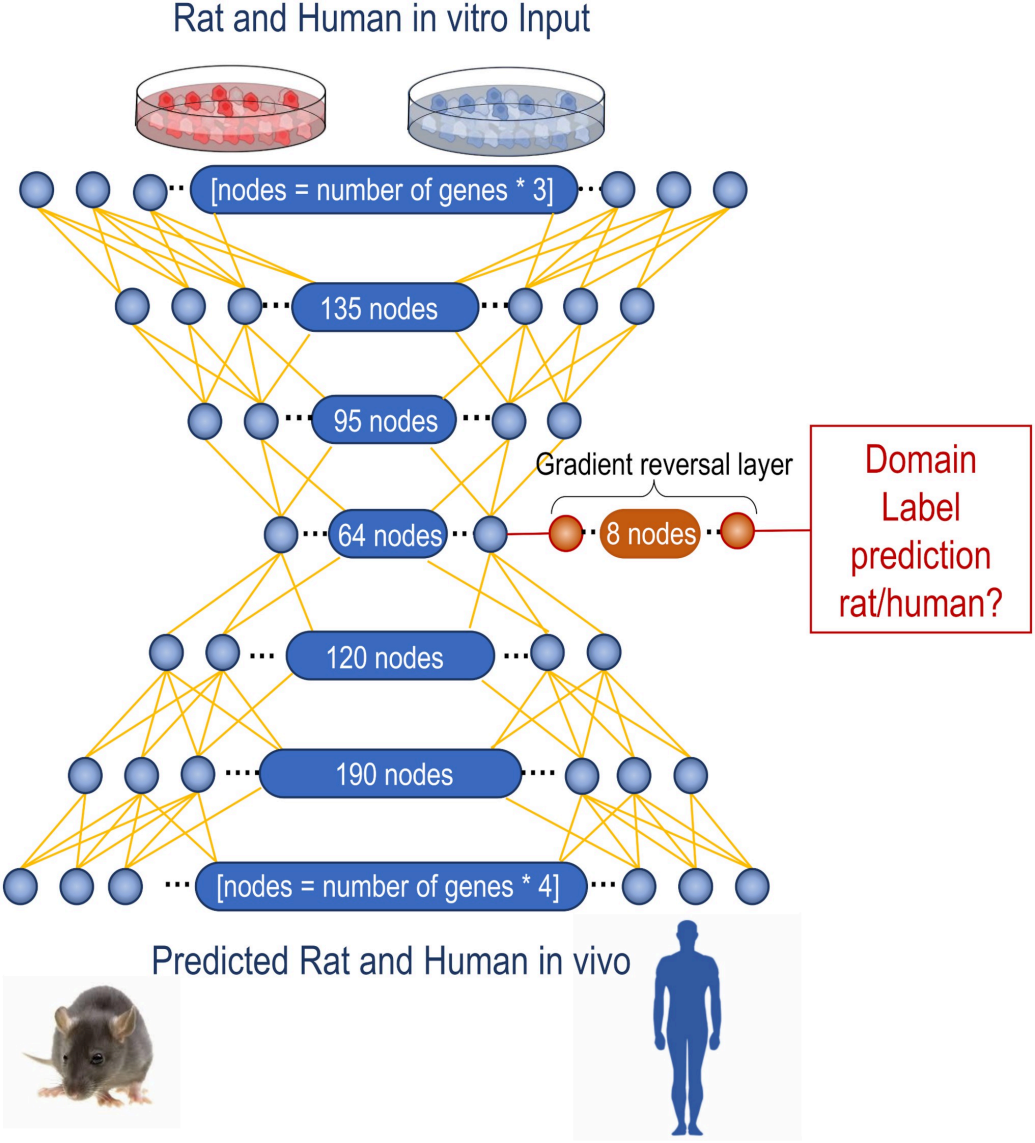
Schema of the implemented unsupervised domain adaptation network. Domain labels are appended to the measured time series of rat (source domain) and human (target domain) *in vitro* gene expression to form the input for the model. The bottleneck architecture of the deep neural network finds a reduced dimension representation of the data. A one-layer classifier is trained to predict the domain labels (rat or human) from the 64-dimensional latent space. The inclusion of a gradient reversal layer in this domain classifier multiples the gradient by a negative scalar in the backpropagation step. Thereby maximising the loss in predicting the domain label. Resulting in domain adaptation as the model cannot discriminate between the source and target domains. Simultaneously the model is trained to reconstruct *in vivo* gene expression patterns using the measured time series of rat *in vitro* gene expression. The network architecture was optimised using a grid search to find a well-performing network. The final network, depicted above, contains five layers consisting of 135, 90, 64, 120, and 190 nodes respectively, with a domain classification layer containing eight nodes. All layers use ReLU activation, except for the output layer, which uses sigmoid activation.

Previous work has demonstrated the ability of deep artificial neural networks (ANNs) with a bottleneck architecture to outperform classical machine learning techniques in translating time series of gene expression from rat to human and from *in vitro* to *in vivo* in rats [17]. Here, Ganin et al.’s unsupervised domain adaptation [26] is applied to the previously validated ANN architecture to train a model to predict human *in vivo* gene expression from measured human *in vitro* gene expression (target domain) using a large labelled data set of rat *in vitro* and *in vivo* gene expression following exposure to a variety of compounds (source domain). Domain adaptation is achieved through the introduction of a domain classification arm from a central hidden layer (Fig 2). The network is trained to maximise the loss in predicting the domain label of the input data (rat or human *in vitro*). The inclusion of a gradient reversal layer, which leaves the input unchanged during forward propagation but reverses the gradient during backpropagation by multiplying the gradient by a negative scalar, allows the domain classifier to be trained in tandem with the *in vivo* prediction using the standard backpropagation algorithm [33]. Through tuning of both the learning rate and the rate at which human data is introduced into the model (lambda), the network constructs a common latent space for the rat and human *in vitro* data which is indiscriminate to the domain of the data.

The structure of the deep neural network was optimised using a grid search. The final network consists of five hidden layers containing 135, 96, 64, 120, 190 nodes respectively (Fig 2). All layers use rectified linear unit (ReLU) activation [34], except for the output layer, which uses sigmoid activation. A single domain classification layer consisting of eight nodes was introduced at the central hidden layer (Fig 2). The gradient reversal parameter lambda increases at a logarithmic rate during training from zero to one, as in the original publication [27]. The domain classification error was calculated using SoftMax cross entropy [27]. The prediction error was calculated as the sum of absolute errors between the measured rat *in vivo* gene expression and the model-predicted gene expression pattern.

The model was trained using Momentum [35], a stochastic-gradient descent algorithm that accelerates convergence to an optimum solution by accumulating gradients from previous steps.

#### Experimental setup

In order to assess how well the model would perform for a previously unseen compound, while still maximising the number of learning examples available for training, leave-one-compound-out cross-validation was used. All sixteen instances for a given compound were removed from the source and target domain. The model was trained on the remaining data. The excluded instances were then used to validate the prediction accuracy of the model. This procedure was repeated for all 45 compounds.

When training the network with domain adaptation, the error term was composed of the sum of the prediction error and domain classification error. The network was also trained without adaptation, in which case the error term consists of just the prediction error. The validation error for each compound-dose combination is the mean absolute error between the model-predicted time series of gene expression and the measured time series of rat *in vivo* gene expression for each gene in the gene set. The overall performance of the model was assessed using the average validation error over all compound-dose instances.

### Latent space classification

The bottleneck structure of the neural network generates a reduced dimensional representation of the time series of both rat and human *in vitro* gene expression data. For example, the GTX+C gene set consists of 76 genes measured at three time points *in vitro*, resulting in a 228-dimensional feature space in the input layer which is reduced to 64 dimensions in the third hidden layer. In recent years, a number of studies have evaluated the reduced dimensional latent spaces generated by variational autoencoders or non-negative matrix factorisation as novel methods for classification in high dimensional genomic data. These low-dimension latent space representations have been successfully utilised to identify subclasses of tumours from RNASeq data [36], predicting drug responses [37], and de-convoluting cell composition of samples [38]. As a result, we decided to explore the 64-dimensional embedding of the rat and human *in vitro* gene expression data in common latent space as a potential method for the classification of toxicity of a novel compound under the assumption that compounds that trigger similar responses in gene expression would cluster together in the compressed latent space. Of the 45 compounds included in this study, the carcinogenicity status (yes/no) is known for 25 compounds [39]. A linear support vector machine (SVM), weighted to account for the unequal number of class labels (19 carcinogenic versus just 6 non-carcinogenic), was trained to discriminate between known carcinogenic and non-carcinogenic compounds using the 64-dimensional embedding of the rat and human *in vitro* gene expression for the low, medium, and high dosages for the labelled compounds during leave-out-out cross-validation. The accuracy of the classification predictions was assessed using the prediction for the labelled leave-one-out compound. This process was repeated to train a predictor of genotoxicity using the 32 compounds for which genotoxicity labels are available [39]. A list of carcinogenicity and genotoxicity labels used for the compounds can be found in S1 Table in S2 File [39].

### Data availability

- All data analysed during the current study are available from the Open TG-GATEs database (https://toxico.nibiohn.go.jp).
- All model scripts are publicly available via a GitHub repository at https://github.com/shauna-odonovan.
- A minimal formatted dataset to reproduce the analysis presented in this study can be found at https://tue.data.surfsara.nl/index.php/s/ABfvy3so7UaOO8V.

## Results

### Domain adaptation

**Fig 3:**
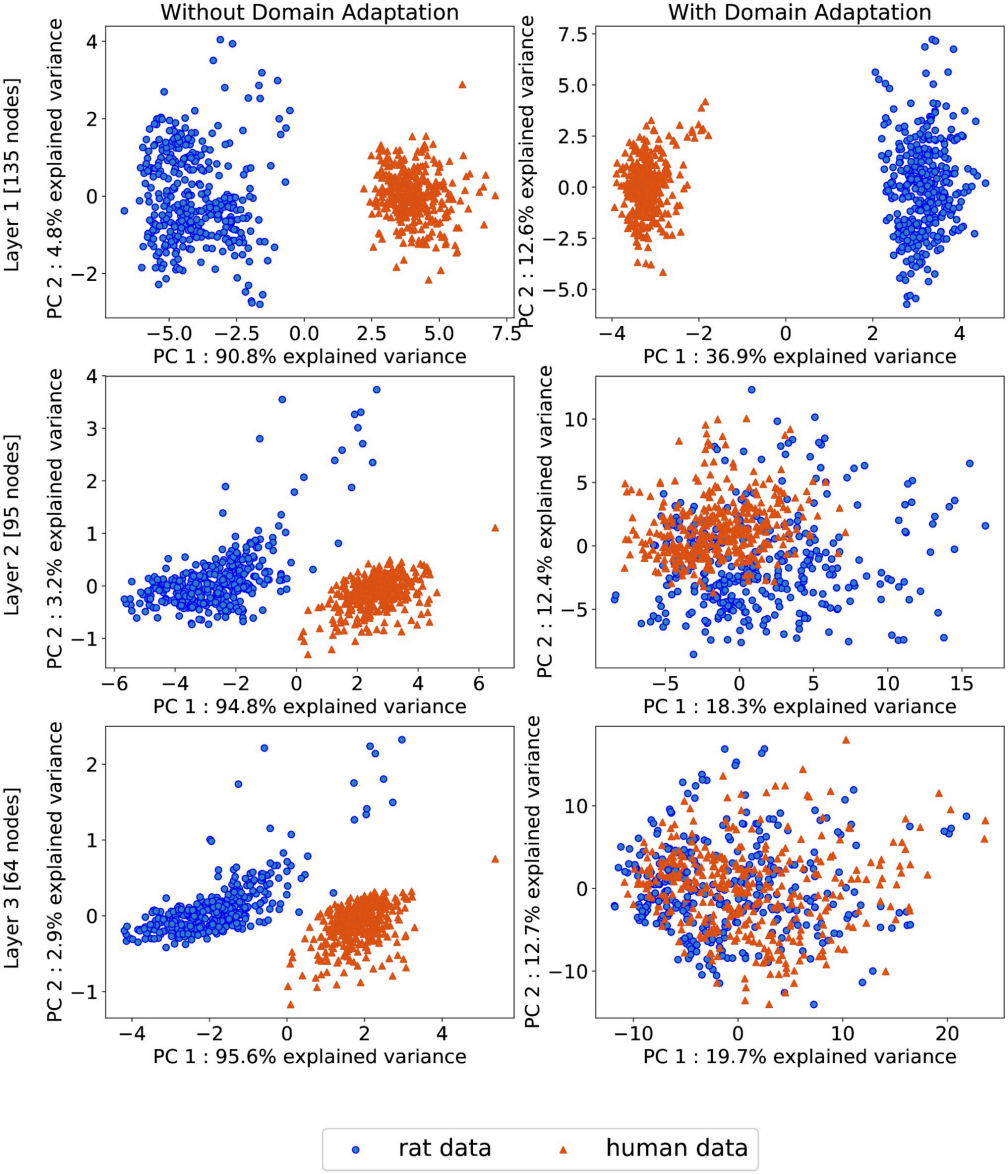
Visualisation of the effect of domain adaptation on the embedding of rat and human data in the network. Visualisation of the embedding of the rat (blue) and human (red) *in vitro* gene expression data in the first three hidden layers of the fully trained network. The first column depicts the embedding of the rat and human *in vitro* data for the network trained without domain adaptation. The second column shows the embedding of the rat and human data for the same network architecture trained with domain adaptation. The first row shows the embedding of each dose-compound combination for both the rat and human *in vitro* data in the first layer of the network, consisting of 135 nodes, projected along the first two principal components of the data. The second and third rows depict the embedding of the rat and human data in the second and third layers of the network respectively. The rat and human data remain disjoint in the first three layers of the network trained without domain adaptation (column one). When the network was trained with domain adaptation the embedding of the rat and human data begin to overlap in the second layer, and are no longer differentiable in the third layer.

Fig 3 demonstrates the effect of domain adaptation on the training of our network. Each row visualises the rat (blue) and human (red) *in vitro* gene expression data along the first two principal components for the embeddings for the first three layers of the network.

The encoding of the rat and human data for the network trained without domain adaptation is depicted in the first column. For this network, the rat and human data are clearly separable along the first principle component in each layer, which captures at least 90% of the variance in the data. The second column details the encoding of the rat and human data in the same network trained with domain adaptation. While the rat and human data are still separable along the first principal component in the first layer, the first principal component explains just 36.9% of the total variance. In the second layer, the border between the rat and human data along the first and second principal components becomes less distinct, with the first component accounting for just 18.3% of the total variance in the data. In the third layer, it is no longer possible to differentiate between the rat and human *in vitro* gene expression instances in the 64-dimensional latent space. The domain classifier predicts the data domain using the encoding of the rat or human data from this third layer. S1 Fig in S1 File illustrates the progress of the domain adaptation during training, showing that at the mid-way point of training the domain adaptation has effectively merged the rat and human data.

### Human *in vivo* predictions

**Fig 4:**
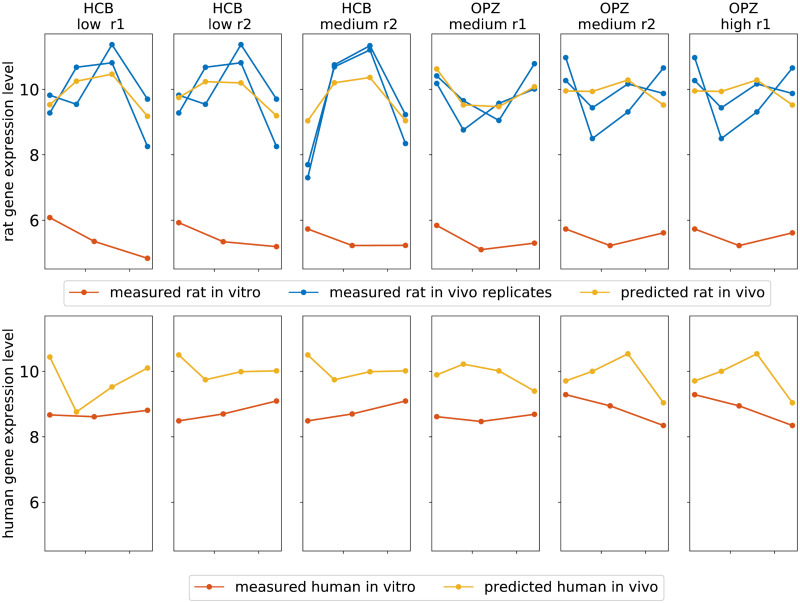
Input data and model predictions for time series of the gene NR0B2 for multiple doses of hexachlorobenzene and omeprazole. The first row depicts the measured time series of rat *in vitro* gene expression (red) and both measured biological replicates of rat *in vivo* gene expression for the gene NR0B2 following exposure to a low (columns 1 and 2) and medium (column 3) dose of hexachlorobenzene (HCB) and a medium (columns 4 and 5) and high dose (column 6) of omeprazole (OPZ). The model prediction of the time series of rat *in vivo* gene expression of NR0B2 for each exposure are shown in yellow. The second row displays the corresponding measured time series of NR0B2 in primary human hepatocytes exposed *in vitro* and the model predicted time series of NR0B2 for the human *in vivo* system.

The model utilises *in vitro* and *in vivo* gene expression data from rats to predict time series of human *in vivo* gene expression given a measured time series of human *in vitro* gene expression following exposure. The network’s human *in vivo* predictions differ in gene expression pattern from the human *in vitro* input and the corresponding measured rat *in vivo* gene expression pattern for a given dose-compound combination, as demonstrated in Fig 4. In addition, the model-predicted gene expression patterns for a given gene are different for different dosages and compounds, as shown in Fig 4 for a sample gene NR0B2, a transcription regulator involved in the regulation of NPAS2-mediated hepatic lipid metabolism that has been associated with hepatic genotoxicity. The model predictions for all 76 genes from the GTX+C gene set following exposure to the previously unseen compounds hexachlorobenzene and omeprazole are shown in S2 and S3 Figs in S1 File No time series of *in vivo* human hepatic gene expression data following exposure to any of the compounds included in this study could be obtained to validate the model predictions.

### Rat *in vivo* predictions

**Fig 5:**
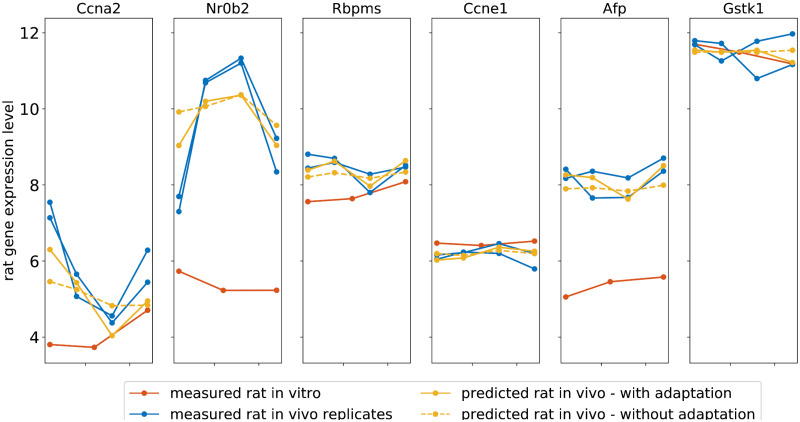
Data and model predictions for a medium dosage of the validation compound hexachlorobenzene for the UDA network trained with domain adaptation for the GTX/C gene set. The figure shows the time series of rat *in vitro* gene expression (red), the input to the model, and both rat *in vivo* biological replicates (blue) measured following an exposure to a medium dosage of hexachlorobenzene for a selection of genes from the GTX/C gene set. The model predictions of rat *in vivo* gene expression pattern for each gene in shown in yellow. The solid yellow line shows predictions of rat *in vivo* gene expression patterns for the network trained with domain adaptation. The dashed yellow lines indicate the predicted rat *in vivo* gene expression patterns for the same network trained without domain adaptation. The rat *in vivo* prediction for the model trained with domain adaptation outperforms those when the model is trained without domain adaptation. When the biological replicates have a similar gene expression pattern the model performs well (Ccna2, Nr0b2, Rbpms, Ccne1). Given the instance matching used to generate machine learning examples in this study, when the biological replicates have contradictory gene expression patterns the model predictions are inaccurate (Afp, Gstk1).

Fig 5 depicts the model predictions for rat *in vivo* gene expression patterns for a selection of genes from the GTX+C gene set for a medium dosage of the previously unseen compound hexachlorobenzene. As before, the measured time series of rat *in vitro* gene expression (model input) is shown in red and the biological replicates of measured rat *in vivo* gene expression are in blue. While the network trained without domain adaptation predicts the general trend of the rat *in vivo* gene expression over the four time points (dashed yellow line), the network trained with domain adaptation captures more of the finer details in the gene expression pattern, out-performing the model trained without domain adaptation (solid yellow line) (Fig 5).

Comparing the average mean absolute error in predicting rat *in vivo* gene expression for the network model trained with and without domain adaptation indicates that the model trained with domain adaptation produces more accurate predictions of rat *in vivo* gene expression for each of the four toxicologically relevant gene sets (Table 1). In fact, the average mean absolute error is significantly lower for the predictions made using the UDA model than using the model without domain adaptation for the Cholestasis, NAFLD, and GTX+C gene sets (p-values < 0.05, using a two-tailed paired t-test).

**Table 1:** Average mean absolute error from leave one out cross validation for the model trained with and without domain adaptation predicting rat *in vivo* gene expression the four toxicologically relevant gene sets identified from literature.

Gene set	Number of genes	with domain adaptation	without domain adaptation	p-value
Cholestasis	18	0.0401 ± 0.0106	0.0409 ± 0.0109	0.0000915*
NAFLD	22	0.0369 ± 0.0071	0.0372 ± 0.007	0.0339*
Steatosis	50	0.0378 ± 0.0055	0.0379 ± 0.0064	0.615
GTX/C	76	0.0337 ± 0.0087	0.0371 ± 0.224	0.00003*

The table shows the average validation error (± one standard deviation) for the network model trained with (column 3) and without domain adaptation (column 4) for each of the four toxicologically relevant genes sets identified from literature. The validation error is the mean absolute error between the measured time series of rat *in vivo* gene expression and the model predicted rat *in vivo* gene expression pattern using leave-one-out cross validation for the 45 compounds included in this study. The fifth column provides the p-value indicating if the difference in average mean absolute error between the network trained with domain adaptation and without domain adaptation are statistically significant, measured using a two-tailed paired t-test. The model trained with domain adaptation has a lower average mean absolute error than the model trained without domain adaptation for all gene sets included in these analyses, the error has significantly decreased for the Cholestasis, NAFLD, and GTX+C gene sets. Column 2 indicates the number of genes included in each gene set.

### Latent space classification

The training accuracy in predicting carcinogenicity using the latent space embedding of rat and human *in vitro* gene expression for genes in the GTX+C gene set of 85.2% indicates that a complete linear separation between carcinogenic and non-carcinogenic compounds within the 64-dimensional latent space of the network is not possible. The accuracy in predicting carcinogenicity for a previously unseen compound using the network trained with domain adaptation is 67.3%. Moreover, the specificity and sensitivity of the method (31.9% and 78.5% respectively) indicate a high false positive rate (Table 2). Predicting genotoxicity labels for a compound using the latent space proves to be a more challenging task. The training accuracy drops to 78.5% for the embedding trained with domain adaptation and 73.3% for the network trained without domain adaptation. The accuracy in predicting genotoxicity status of a previously unseen compound is just 51%.

**Table 2:** Training and validation accuracy in classifying carcinogenicity and genotoxicity of a compound from latent space embedding of rat and human *in vitro* gene expression data.

GTX+C gene set	Training accuracy	Validation accuracy	sensitivity	specificity
Carcinogenicity (n = 15)				
With domain adaptation	0.0852	0.673	0.785	0.319
Without domain adaptation	0.807	0.590	0.741	0.111
Genotocxicity (n = 31)				
With domain adaptation	0.785	0.510	0.299	0.638
Without domain adaptation	0.733	0.559	0.222	0.763

The first section of the above table lists the training and validation accuracies for a linear support vector machine trained to predict carcinogenicity status of a previously unseen compound using the 64 dimensional embedding of the rat and human *in vitro* gene expression from the third hidden layer of the neural network trained for the GTX+C gene set using leave-one-out cross validation. The linear SVM was trained to predict carcinogenicity for the latent space from the latent space embedding networks trained both with domain adaptation (row 1) and without domain adaptation (row 2). The lower section presents the training and validation errors for the linear SVM trained to predicting genotoxicity from the latent space embeddings of the rat and human *in vitro* gene expression data.

## Discussion

In recent years toxicogenomic assays have achieved notable success in predicting the hepatotoxicity of a novel compound [4–13]. However, relating changes in gene expression profiles from these rodent and cell line assays to relevant human outcomes still proves challenging. Here, we applied transfer learning to leverage a large publicly available database of *in vitro* and *in vivo* gene expression in rats to train a deep learning model to predict human *in vivo* gene expression. We demonstrate that this method has successfully achieved domain adaptation, with the rat and human data being indiscriminate in the network latent space. Moreover, the inclusion of the human *in vitro* data significantly improved the accuracy of the rat *in vivo* gene expression predictions.

Incorporating human *in vitro* gene expression data in the domain adaptation network significantly improves the accuracy of the rat *in vivo* gene expression predictions for three of the four toxicologically relevant gene sets included in this analysis, illustrated in Fig 5. While the network trained without domain adaptation predicts the general trend in the gene expression pattern (Fig 5, dashed yellow lines Ccena2, Nr0b2, Rbpms, and Afp) the improvement in prediction accuracy for the network trained with domain adaptation is evident, with the solid yellow lines more closely predicting the dynamics in measured time series of gene expression. The network trained without domain adaptation was optimised solely to predict the time series of rat *in vivo* gene expression. Therefore, it was somewhat unexpected that training the network to predict both the rat *in vivo* gene expression and the domain label would result in a significantly improved prediction of rat *in vivo* gene expression. We postulate that incorporating the human *in vitro* data introduces additional, relevant information, serving as a form of regulation for the rat *in vitro* to rat *in vivo* prediction task preventing overfitting.

The deep learning network produces physiologically plausible predictions of human *in vivo* gene expression given a measured time series of human *in vitro* gene expression following exposure to a previously unseen compound. However, given the invasive nature of sampling the liver, no relevant measured time series of human *in vivo* gene expression data could be obtained to validate our model predictions. Nevertheless, the model predicted human *in vivo* gene expression patterns differ from the time series of human *in vitro* gene expression. Demonstrating that the model is not simply reproducing the input it receives. Moreover, the human *in vivo* predictions also differ from the rat *in vivo* predictions for the same gene, indicating that the model is not simply predicting the same output for both the rat and human. In addition, the predicted human *in vivo* gene expression pattern for a given gene alters for different exposure conditions (dosages and compounds), demonstrating that the model is not predicting the same human *in vivo* gene expression pattern in all instances for a given gene. While we do not have the data to validate the quality of our human *in vivo* predictions, Ganin et al.’s method for domain adaptation was shown to outperform the state-of-the-art algorithms in image recognition and natural language processing In the future, should relevant human *in vivo* data become available our model predictions could be validated. Moreover, Ganin et al.’s method can be easily generalised to semi-supervised learning, allowing even sparse human *in vivo* data to be integrated into training to improve human *in vivo* predictions [27].

Recent studies have reported some success in separating subclasses of tumours from the latent space embedding of tumour-derived RNA-Seq data [36] or identifying processes involved in rare diseases from a reduced dimension latent space trained using large publicly available databases of gene expression data [40]. While our network was trained to predict time series of rat *in vitro* gene expression and not carcinogenicity or genotoxicity status, we hypothesised that compounds that induce a carcinogenic or genotoxic phenotype would trigger similar temporal gene expression responses and would therefore cluster together in the reduced dimension common latent space. Consequently, the embedding of the rat and human *in vitro* gene expression data in the 64-dimensional common latent space was explored as a potential method for the classification of toxicity of a novel compound. Our analysis indicated it was not possible to get a complete linear separation between carcinogenic and non-carcinogenic compounds in the common latent space generated by our model, with an accuracy of just 67.3% in predicting the carcinogenicity status of a previously unseen compound. The latent space classification falls short of existing classification methods which achieve over 80% accuracy in classifying carcinogenicity in combination with the Ames mutagenicity assay [6]. Classifying genotoxicity proved to be more difficult, with the SVM trained on the latent space representation achieving an accuracy of just 51% in predicting the genotoxicity status for a previously unseen compound. As it currently stands, the reduced dimensional common latent space generated by our models does not appear to be a viable novel method for the classification of compound toxicity. However, our network has been trained using just 720 learning examples of gene expression for just 76 genes (GTX+C gene set). Green and Way trained their variational autoencoder to reconstruct transcriptomic profiles of 5,000 genes using RNASeq data for 10,459 tumour and tumour-adjacent normal samples from The Cancer Genome Atlas [36]. In future work, the classification accuracy of the reduced dimensional latent space could be improved by integrating gene expression data from other data sets, increasing the number of learning examples and, consequently, the number of genes that can be included.

The open TG-GATEs dataset was selected for this study as it is a comparatively large dataset containing gene expression profiles for *in vitro* and *in vivo* rat as well as human *in vitro* following exposure to a range of compounds. In order to maximise the number of learning examples available to train our model we elected to consolidate data from the 45 available compounds. While supplementing the data set with data from other sources would have further increased the number of learning examples, it would also introduce additional variation between the data due to differing experimental protocols that may negatively impact the domain adaptation. Consequently, we decided to restrict our analysis to just the TG-GATEs data set. Nevertheless, the addition of gene expression profiles from other toxicological databases, such as DrugMatrix [19], may improve the accuracy of the model predictions of gene expression and classification of compounds using the reduced dimensional common latent space. The UDA approach proposed by Gannin et al. provides a highly flexible way to achieve domain adaptation within the context of a generalisable neural network architecture and has been successfully applied to a number of challenges including image processing [41] and natural language processing [27]. In this study, we apply UDA to gene expression data from different species. In Fig 3 we show that without domain adaptation the rat and human data cannot be combined and occupy distinct regions of the latent space, however when the network is trained with domain adaptation the rat and human data are indiscriminable. Moreover, we show that the incorporation of human data significantly improves the temporal accuracy of the rat *in vivo* gene expression prediction. In the future this generalisable UDA architecture could prove to be a valuable tool for the improved integration of data from multiple sources or data platforms, correcting for batch effects or variation in experimental protocol between the data sets. UDA may also facilitate the training of predictor models for limited data sets of human *in vivo* data by leveraging larger, publicly available data sets from *in vitro* and animal studies.

The domain adaptation network was trained using Momentum, a stochastic gradient descent algorithm. The gradient reversal parameter (lambda) increased from 0 to 1 at a logarithmic rate, as suggested by Ganin et al. [27]. When optimising the network architecture it was observed that the domain classifier is highly susceptible to mode collapse, whereby the network maximises the loss in predicting the domain of the input data by simply predicting human for every case. This may be due to the limited number of learning examples, as mode collapse was more prevalent when training the network for the larger gene sets. The frequency of mode collapse was reduced by placing a lesser weight on the domain classification error during the early phase of training. In instances when mode collapse occurred, the network was retrained. We implement the domain adversarial training of a neural network as proposed by Ganin et al. which necessitated the introduction of a gradient reversal layer to allow the maximisation of loss in the domain prediction to be trained in tandem with the *in vivo* prediction. As we use data with binary labels (rat or human) we hypothesise that minimising the loss on predicting the incorrect domain label would achieve the same effect on training the network as maximising the loss on predicting the correct domain label without the need for the gradient reversal layer.

To conclude, we have successfully applied domain adaptation in the context of a deep neural network; merging rat and human gene expression data to facilitate the prediction of human *in vivo* gene expression using a large, labelled set of rat data. Incorporation of the human *in vitro* gene expression data when training the network significantly improves the accuracy of the predictions of rat *in vivo* gene expression patterns following exposure to a previously unseen compound. The ability of the reduced dimensional common latent space generated by our network to discriminate between sub-classes of toxicity was comparable to existing methods for compound classification. In future work, we anticipate that with sufficient learning examples the reduced dimension latent space trained in our network would outperform existing methods for compound toxicity classification.

## Supporting information

S1 File Additional figures. (PDF)

S2 File Data information. List of subset of compounds from open TG-GATEs and toxicologically relevant gene lists identified from literature included in these analyses.
(PDF)

S1 Data All model scripts are publicly available from GitHub repository (https://github.com/shauna-odonovan). (ZIP)
