# Supplementary material for: Application of transfer learning to predict drug-induced human in vivo gene expression changes using rat in vitro and in vivo data
Source: PLoS One. 2023 Nov 30;18(11):e0292030. doi: 10.1371/journal.pone.0292030 (PMC10688741; doi:10.1371/journal.pone.0292030)
Supplement: S1 File — (PDF) [file pone.0292030.s001.pdf]

Section 2 Supplementary figures accompanying;

"Application of transfer learning to predict drug-induced human in vivo gene expression changes using rat in vitro and in vivo data"

O'Donovan SD, Cavill R, Wimmenauer F, Lukas A, Stumm T, Smirnov E, Lenz M, Ertaylan G, Jennen DGJ, van Riel NAW, Driessens K, Peeters RLM, de Kok TMCM.

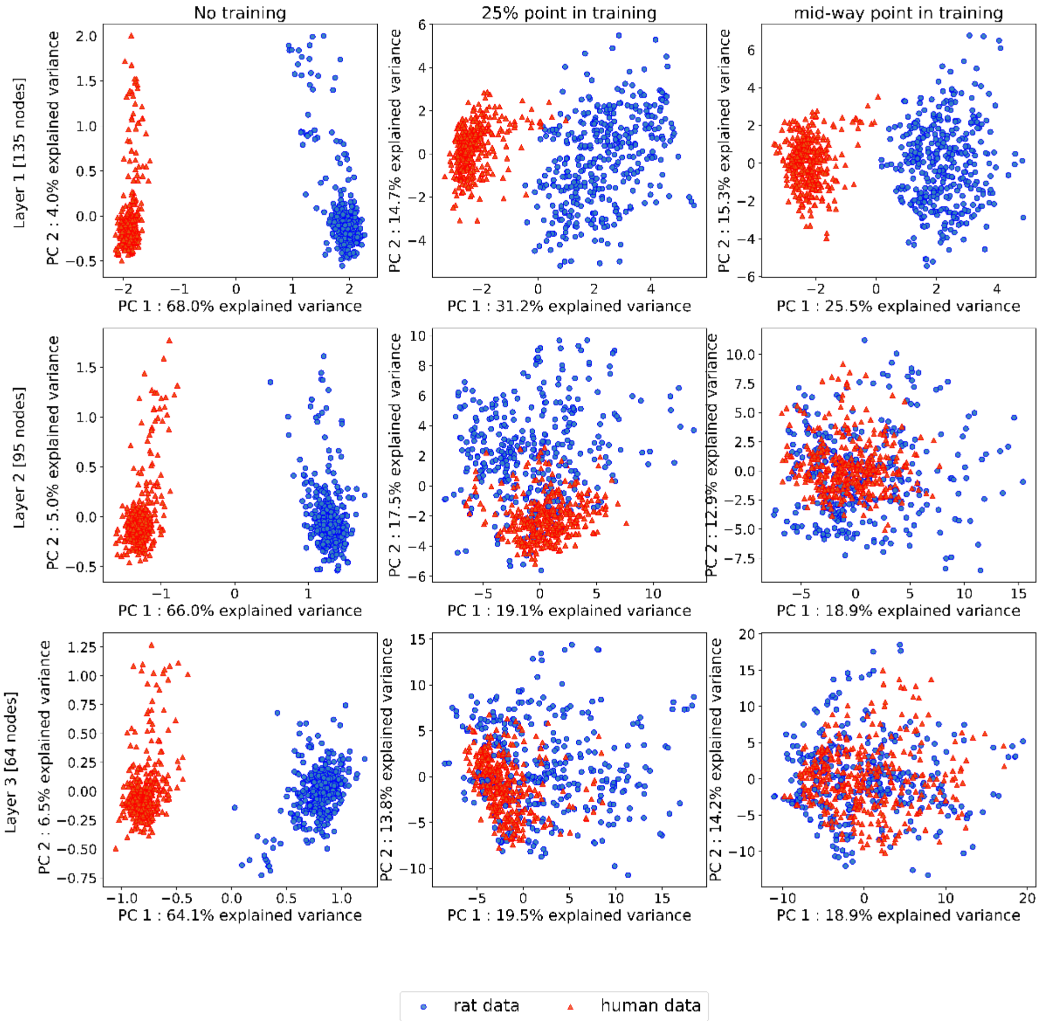

Supplemental figure S1: Impact of domain adaptation on the network during training
Visualisation of the effect of domain adaptation on the embedding of the source data (rat gene expression (blue)) and the target domain data (human gene expression (red)) during training. Each row shows a two dimensional projection of the embedding of the data along the first two principal components for the first three layers of the network. The first column shows the initial embedding of the rat and human data in the network before training. The rat and human data occupy disjoint regions of the latent space. The second column depicts the embedding of the rat and human data one quarter for the way through training. The difference in the distributions of the rat and human data becomes less distinct. The final column shows the embedding of the rat and human data mid-way through training. At this point the domain adaptation has effectively merged the rat and human data

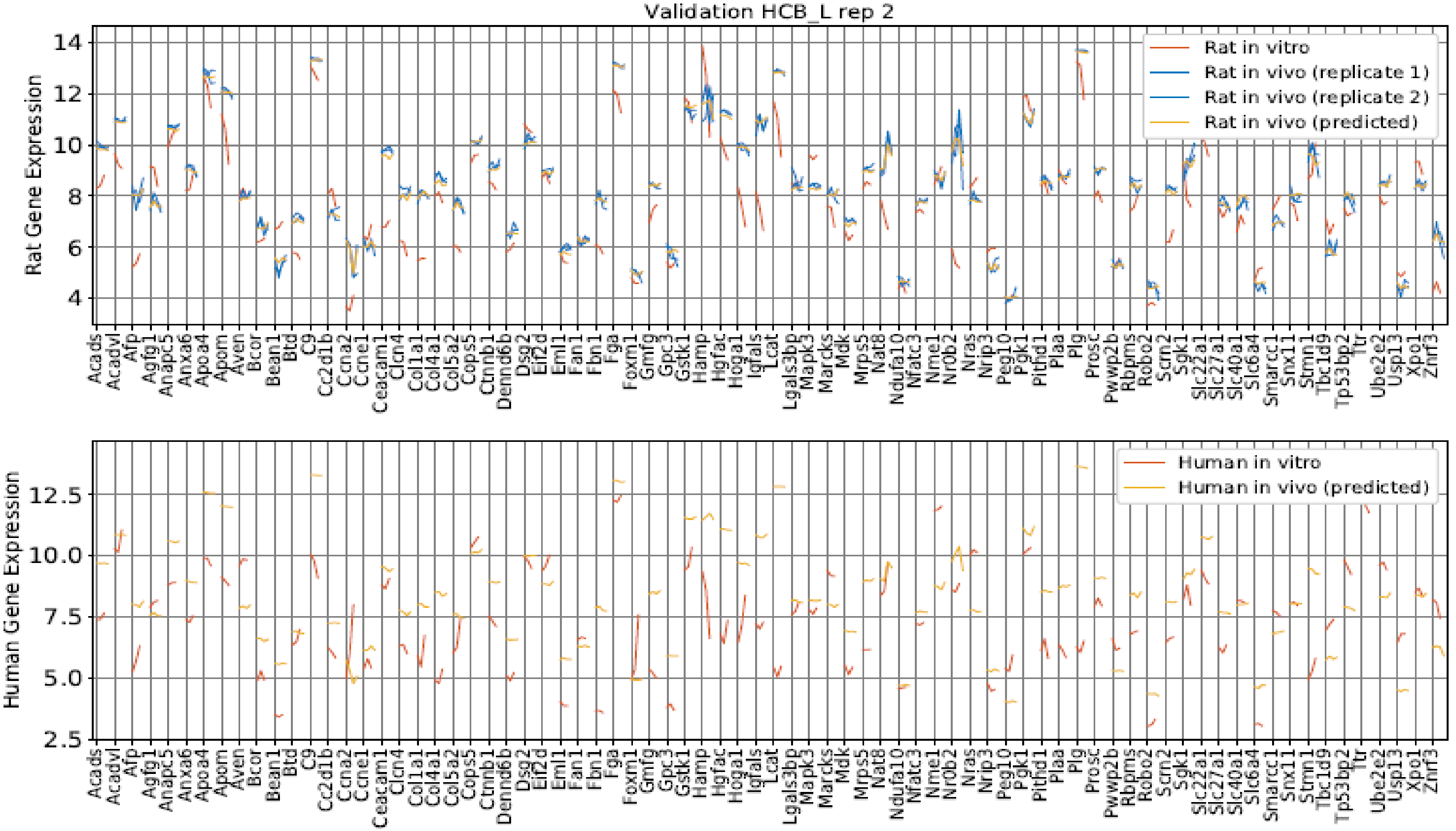

Supplemental Figure S2: Measured and model predicted rat and human gene expression for GTX/C gene set for a low dose of hexachlorobenzene
The upper panel depicts the data from the source domain. The measured rat \textit{in vitro} gene expression (modedl input) for the 76 gene in the GTX/C gene set are shown in red and both rat \textit{in vivo} biological replicates in blue. The model rat \textit{in vivo} predictions are shown in yellow. The lower panel shows corrisponding target data. The measured human \textit{in vitro} is shown in red and model human \textit{in vivo} predictions in yellow.

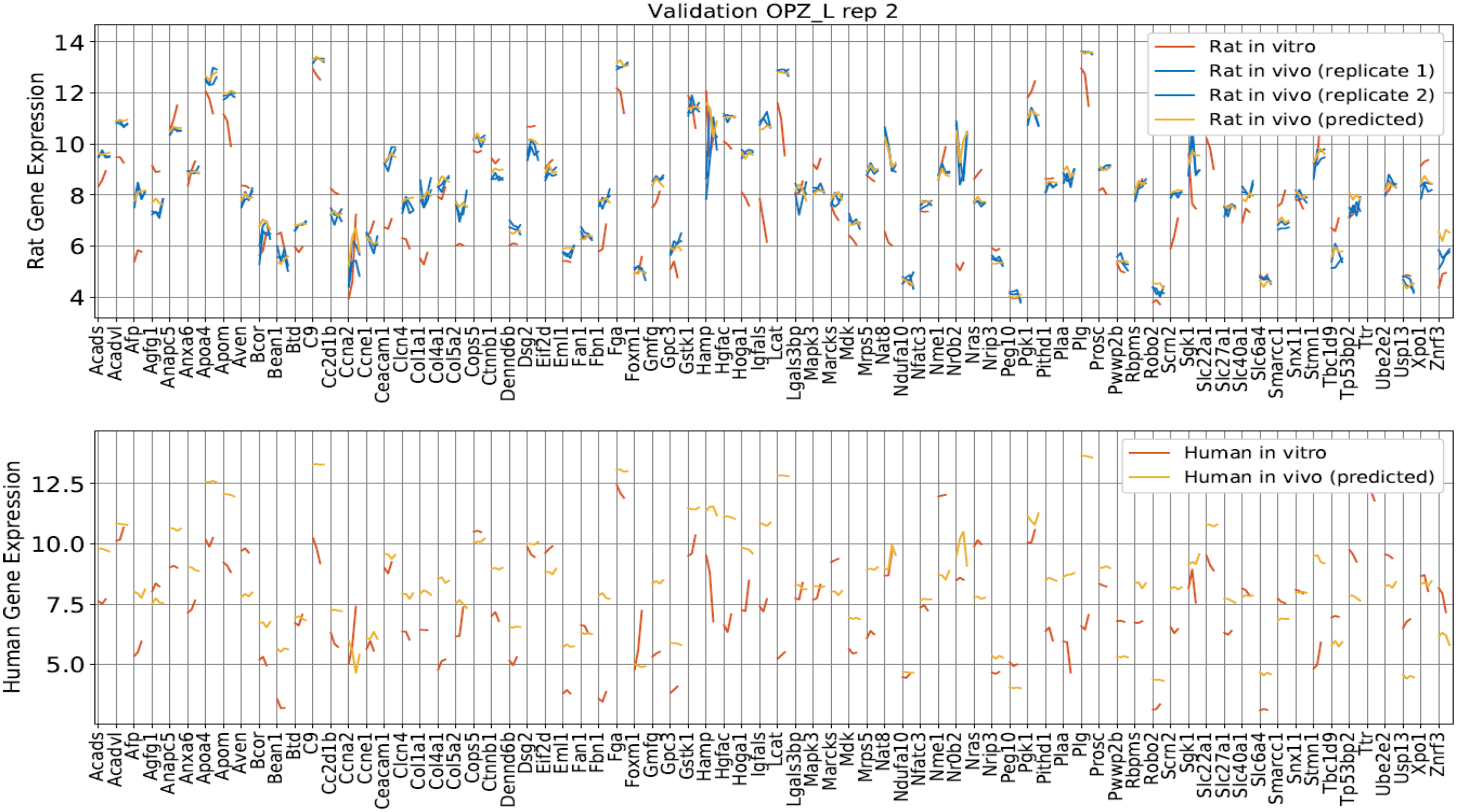

Supplemental Figure S3: Measured and model predicted rat and human gene expression for GTX/C gene set for a low dose of omeprazole.
The upper panel depicts the data from the source domain. The measured rat \textit{in vitro} gene expression (input to the mode) for the 76 gene in the GTX/C gene set for the previously unseen compound hexachlorobenzene are shown in red and both rat \textit{in vivo} biological replicates are in blue. The model predictions of rat \textit{in vivo} gene expression are shown in yellow. The lower panel shows corresponding target data. The measured human \textit{in vitro} is shown in red and model predictions of human \textit{in vivo} gene expression are in yellow.
