# Supplementary material for: Application of transfer learning to predict drug-induced human in vivo gene expression changes using rat in vitro and in vivo data
Source: PLoS One. 2023 Nov 30;18(11):e0292030. doi: 10.1371/journal.pone.0292030 (PMC10688741; doi:10.1371/journal.pone.0292030)
Supplement: S2 File — List of subset of compounds from open TG-GATEs and toxicologically relevant gene lists identified from literature included in these analyses. (PDF) [file pone.0292030.s002.pdf]

Section 2 Supplementary material accompanying;

“Application of transfer learning to predict drug-induced human in vivo gene expression changes using rat in vitro and in vivo data”

O’Donovan SD, Cavill R, Wimmenauer F, Lukas A, Stumm T, Smirnov E, Lenz M, Ertaylan G, Jennen DGJ, van Riel NAW, Driessens K, Peeters RLM, de Kok TMCM.

Table S1 :Subset of compounds from TG-GATEs included in this analysis [1,2].

compound	abbreviation	Carcinogenicity	Genotoxicity
Acetaminophen	APAP	C	GTX
Adapin	ADP	unknown	unknown
Allopurinol	APL	unknown	GTX
Allyl alcohol	AA	unknown	unknown
Aspirin	ASA	NC	NGTX
Azathioprine	AZP	C	GTX
Benzbromarone	BBr	unknown	unknown
Bromobenzene	BBZ	unknown	NGTX
Carbamazepine	CBZ	unknown	unknown
Chlorpromazine	CPZ	unknown	NGTX
Cimetidine	CIM	NC	unknown
Clofibrate	CFB	C	GTX(*textit{in vitro}*), NGTX(*textit{in vivo}*)
Coumarin	CMA	C	GTX(*textit{in vitro}*), NGTX(*textit{in vivo}*)
Cyclophosphamide	CPA	C	GTX
Diazepam	DZP	NC	GTX
Diclofenac	DFNa	NC	NGTX
Ethionine	ET	unknown	unknown
Fluphenazine	FP	unknown	GTX
Flutamide	FT	unknown	unknown
Gemfibrozil	GFZ	unknown	unknown
Glibenclamide	GBC	unknown	unknown
Griseofulvin	GF	C	GTX(*textit{in vitro}*), NGTX(*textit{in vivo}*)
Haloperidol	HPL	unknown	NGTX
Hexachlorobenzene	HCB	C	GTX

Indomethacin	IM	unknown	unknown
Isoniazid	INAH	C	GTX
Ketoconazole	KC	unknown	unknown
Labetalol	LBT	unknown	NGTX
Lomustine	LS	C	GTX
Methapyrilene	MP	unknown	GTX
Methyltestosterone	MTS	unknown	unknown
Naphthyl isothiocyanate	ANIT	unknown	NGTX
Nitrofurantoin	NFT	C	GTX(\textit{in vitro}), NGTX(\textit{in vivo})
Omeprazole	OPZ	unknown	NGTX
Perhexiline	PH	unknown	unknown
Phenobarbital	PB	C	GTX(\textit{in vitro}), NGTX(\textit{in vivo})
Phenylbutazone	PhB	C	GTX(\textit{in vitro}), NGTX(\textit{in vivo})
Phenytoin	PHE	C	NGTX
Propylthiouracil	PTU	C	NGTX
Rifampicin	RIF	unknown	unknown
Sulfasalazine	SS	C	NGTX
Tetracycline	TC	C	GTX
Thioacetamide	TAA	C	GTX
Thioridazine	TRZ	unknown	unknown
Valproic acid	VPA	unknown	NGTX
WY-14643	WY	C	NGTX

Overview of gene sets included in these analyses

Table S2: Cholestasis gene set identified from literature [3-5]

HNF4A	SLC10A1	SLCO1B1	CYP7A1	CYP8B1
CYP27A1	CYP7B1	NR1H4	NR0B2	N21L2
NR1L3	FGF19	ABCB11	SLC51A	ABCC3
	UGT2B4	CYP3A4	SULT2A1	

Table 3: NAFLD gene set identified from literature [6].

PPARGC1A	IL6	SERPIN1	IL1B	STAT3	TCF7L2
PNPLA3	PEMT	TM6SF2	SREBF1	HFE	SAMM50
FDFT1	NR1I2	PPARA	PPP1R3B	CHDH	LYPLAL1
		SOD2	LEPR		

Table 4: Steatosis gene set; generated in house.

FABP4	ACACA	AKT1	AKT2	AKT3	PRKAA1	PRKAA2	ADIPOR1
ADIPOR2	ADIPOQ	BCL2A1	CPT2	CPT1A	CPT1C	CASP8	MLXIPL
FABP5	ELOVL3	FAS	FOXO1	NR1H4	RXRA	FASLG	FABP3
FABP7	PMP2	GCKR	IL1A	IL10	IRS1	IRS2	MAPK10
NFKB1	NFKB2	RELA	RELB	PPARA	PPARG	PTEN	RXRB
RXRG	SCD	SOCS3	SREBF1	TGFB1	TGFB2	TGFB3	TLR4
		MTOR			PNPLA3		

The steatosis gene set was generated in house combining a literature search using the search terms “liver steatosis”, “Nonalcoholic fatty liver disease”, and “NAFLD”, the steatosis pathway from KEGG (hsa04932) and the steatosis adverse outcome pathway from Wikipathways. In addition genes were filtered to include only known human –rat orthologs measured by both the Rat Genome 230 2.0 Array and the Human Genome U133 Plus 2.0 Array used in this study. This gave rise to a seed gene set of 45 genes, these were then used an input for input for MetaCore (version 6.30, build 68780, accessed on 9th of May 2017) to generate a fully connected gene interaction network. Dijkstra's shortest path algorithm is used to construct the network with allowing one node to be added if necessary. This analysis yielded the final set of 50 genes.

Table 5: gene set reported as being a genomic signature of genotoxicity/carcinogenicity [7,8].

CEACAM1	CLCN4	EML1	PWWP2B	UBE2E2	USP13	GMFG
PROSC	TTR	NR0B2	NAT8	RBPMS	TBC1D9	SNX11
BCOR	ROBO2	DENND6B	APOM	NR1P3	PITHD1	AVEN
ZNRF3	BEAN1	SLC27A1	ANXA6	APOA4	BTD	EIF2D
AGFG1	NDUFA1-	NFATC3	PLAA	FAN1	SLC40A1	ANAPC5
MRPS5	GSTK1	HOGA1	FGA	SGK1	SLC6A4	SCRN2
CC2D1B	GPC3	MDK	COL5A2	TP53BP2	XPO1	AFP
CCNA2	CCNE1	COL1A1	COL4A1	CTNNB1	FBN1	FOXM1
STMN1	LGALS3BP	MARCKS	NME1	NRAS	PGK1	MAPK3
SMARCC1	COPS5	PEG10	HGFAC	IGFALS	LCAT	SLC22A1

ACADS	ACADVL	C9	DSG2	PLG	HAMP

References

1. Igarashi Y, Nakatsu N, Yamashita T, et al. Open TG-GATEs: a large-scale toxicogenomics database. *Nucleic Acids Res*. 2014;43(Database issue):D921-7.
2. Nair SK, Eeles C, Ho C, Beri G, Yoo E, Tkachuk D, Tang A, Nijrabi P, Smirnov P, Seo H, Jennen D, Haibe-Kains B. ToxicoDB: an integrated database to mine and visualize large-scale toxicogenomic datasets. Nucleic Acids Res. 48(W1):W455-W462. (2020)
3. Jung, D., Elferink, M. G. L., Stellaard, F., Groothuis, G. M. M.. "Analysis of bile acid-induced regulation of FXR target genes in human liver slices," *Liver Int.*, 27(1), 137–144 (2007).
4. Vinken, M., Landesmann, B., Goumenou, M.,Vinken, S., Shah, I., Jaeschke, H. et al. "Development of an Adverse Outcome Pathway From Drug-Mediated Bile Salt Export Pump Inhibition to Cholestatic Liver Injury," *Toxicol. Sci.*, 136,. 97–106, (2013).
5. Liu, J., Lu, H.. Lu, Y.-F., Lei, X., Cui,. Ellis, J. Y E et al. "Potency of individual bile acids to regulate bile acid synthesis and transport genes in primary human hepatocyte cultures.," *Toxicol. Sci.*, 141, 538–46 (2014).
6. Ryaboshapinka M, Hammar M. "Human hepatic gene expression signature of non-alcoholic fatty liver disease progression, a meta-analysis." *Scientific Reports* 7:12361(2017)
7. Magkoufopoulou, C., Claessen, S.M.H., Tsamou, M., Jennen, D.G.J., Kleinjans, J.C.S., van Delft; J.H.M. "A transcriptomics-based *\textit{in vitro}* assay for predicting chemical genotoxicity *\textit{in vivo}",Carcinogenesis*, 33, 1421–1429,(2012)
8. Caiment, F., Tsamou, M., Jennen, D., Kleinjans J. Assessing compound carcinogenicity *\textit{in vitro}* using connectivity mapping, *Carcinogenesis,* 35, 201–207, (2014)
