# Supplementary material for: Application of transfer learning to predict drug-induced human in vivo gene expression changes using rat in vitro and in vivo data
Source: PLoS One. 2023 Nov 30;18(11):e0292030. doi: 10.1371/journal.pone.0292030 (PMC10688741; doi:10.1371/journal.pone.0292030)
Supplement: S1 Data — (ZIP) [file pone.0292030.s003.zip › Unsupervised_Domain_Adaptation_Code/READ_ME.pdf]

Domain_Adaptation_Gene_Expression

Application of unsupervised domain adaptation to train a model to predict human in vivo gene expression following exposure to a previously unseen compound using the rat in vitro, rat in vivo, and human in vitro gene expression from the open TG-GATEs data set

This project is the code refernced to in "Application of transfer learning to predict drug-induced human in vivo gene expression changes using rat in vitro and in vivo data" by Shauna O'Donovan, Rachel Cavill, Florian Wimmenauer, Alexander Lukas, Tobias Stumm, Evgueni Smirnov, Michael Lenz, Gokhan Ertaylan, Danyel Jennen, Natal van Riel, Kurt Driessens, Ralf Peeters, Theo de Kok.

Dependancies The scripts are implemented in Python 3, tensorflow V1 is required to run the scripts. Versions are specified in the jupyter notebook "RUN_MODEL"

How to run Sample data files can be downloaded from the following link.

https://surfdrive.surf.nl/files/index.php/s/dyVphSI1xXS8Zxi

This folder contains three files containing processed rat in vitro, human in vitro, and rat in vivo gene expression data obtained from open TG-GATEs [1]. The original raw data micro-array data can be downloaded in the form of CEL files from https://toxico.nibiohn.go.jp. The micro-array data has been pre-processed using Affymetrix Power Tools using the robust multi-array average normalisation method and stored in the form of pickle files as follows:

data[gene][compound][dosage][replicate][time]

Where genes are indicated by name (gene symbol). Compound, dosage, replicate, and time can be indicated by index.

To train the model with domain adaptation according to the approach of Ganin et al [2] for using provided toxicologicaly relevent gene sets (i.e. NAFLD, STEATOSIS etc.) run the script titled "Train_Model_with_Domain_Adaptation.py". This will train the model using leave one out cross validation for all 45 compounds present in the dataset. The model output will be saved in the specified folder in the "export" folder. Data on intermediate steps of training will be stored in the "midterm" folder. Plots of the final predictions will be saved in the "plots" folder. It is also possible to train the network without the domain adaptation step using the "Train_Model_without_Domain_Adaptation.py" script.

#References

1. Igarashi, Y., Nakatsu, N., Yamashita, O, Ono, A., Urushidani, T., Yamada, H., Open TG-GATEs: a large-scale toxicogenomics database. Nucleic Acids Res. 43, 21-7.
2. Ganin Y, Ustinova E, Ajakan H, Germain P, Larochelle H, Laviolette F,Marchand M, Lempitsky V. Domain-adversarial training of neural networks. J.Mach. Learn. Res. 17, 1–35. (2016).
